# Sedation in Critically Ill Children

**DOI:** 10.3390/jcm14176273

**Published:** 2025-09-05

**Authors:** Stephen Playfor, Lara Bunni

**Affiliations:** Paediatric Intensive Care Unit, Royal Manchester Children’s Hospital, Oxford Road, Manchester M13 9WL, UK; lara.bunni@mft.nhs.uk

**Keywords:** pediatric intensive care (PICU), sedation, analgesia, tolerance, withdrawal, delirium

## Abstract

Sedation and analgesia are crucial elements in managing discomfort and facilitating critical care interventions in children. Our choice of sedative agents has a significant impact on the physiological and psychological outcomes of our patients. Oversedation and undersedation are associated with adverse events, including increased risk of Pediatric Intensive Care Unit (PICU) readmission, mortality, and longer duration of mechanical ventilation. Studies have shown significant variation in sedation and analgesia practices across different regions and specialties. Consensus clinical guidelines have been developed to standardize sedation and analgesia practices; commonly used intravenous agents include opioids (fentanyl, morphine, and remifentanil), α-2 agonists (clonidine and dexmedetomidine), benzodiazepines (particularly midazolam), ketamine, and volatile anesthetic agents (isoflurane and sevoflurane). Our goal should be to administer the smallest possible number of sedative and analgesic agents, in the lowest possible doses, for the shortest amount of time, whilst adequately controlling the pain and agitation of our patients. Aside from drug management, non-pharmacological interventions, such as family presence, music, and virtual reality, can also play a significant role in maintaining comfort in critically ill children. Validated clinical tools are available to measure sedation and to assess iatrogenic withdrawal syndrome and delirium. Daily interruption of sedatives and protocolized sedation management has been associated with a reduction in the duration of mechanical ventilation and length of PICU admission in some studies, but their effectiveness is still debated. Further research is needed to optimize sedation and analgesia practices in critically ill children. By adopting evidence-based guidelines and incorporating non-pharmacological interventions, clinicians may be able to improve patient outcomes and also reduce the incidence of adverse events.

## 1. Introduction

Sedation and analgesia are some of the most common treatments administered to critically ill children worldwide to manage their discomfort and to facilitate critical care interventions. These agents are often considered as inconsequential treatments, administered to enable life-saving management. It is increasingly clear, however, that the choice of sedative agents has a significant impact on the physiological and psychological outcomes of our patients with both oversedation and undersedation being associated with significant adverse events. Ding and colleagues have demonstrated that the administration of any sedative agents during Pediatric Intensive Care Unit (PICU) admission is a significant risk factor for PICU readmission within 1 year of discharge (62.14% vs. 48.05%, *p* = 0.047) [1]. In adult patients, deeper levels of sedation have been linked to higher rates of mortality (interquartile odds ratio (OR) = 5.42, 4.23–6.95; *p* < 0.001) and a significant reduction in days free from mechanical ventilation (−7.27; *p* < 0.001), ICU-free days (−4.38; *p* < 0.001), and hospital-free days (−7.00; *p* < 0.001) [2]. In a propensity-matched retrospective cohort study of critically ill adult surgical patients, Wu and colleagues demonstrated that the use of deeper levels of sedation were associated with increased mortality a year after hospital discharge [3]. In 2013, Shehabi and colleagues demonstrated that deep sedation in critically ill adults was a factor independently associated with prolonged duration of mechanical ventilation (hazard ratio (HR) 0.93, 95%, confidence interval (CI) 0.89–0.97, *p* = 0.003), death in hospital (HR 1.11, 95% CI 1.05–1.18, *p* < 0.001), and mortality at 180 days (HR 1.09, 95% CI 1.04–1.15, *p* = 0.002) [4]. Conversely, Treggiari and colleagues have demonstrated that a strategy of light sedation in critically ill adult patients is associated with benefits in terms of a reduced duration of critical care admission and length of mechanical ventilation without there being any negative impacts on the mental health of patients or the number of patient safety events [5]. Throughout this review, for clarity, any references to the provision of sedation will generally apply to the administration of combinations of sedative and analgesic agents as ‘sedation’ instead of the more technically correct term ‘analgo-sedation’.

Patel and colleagues published a paper in 2020 evaluating sedation, analgesia, and the administration of neuromuscular blocking agents in 66,443 critically ill pediatric patients between 2009 and 2016 in 161 US PICUs [6]. The authors documented that 63.3% of children (42,070) received analgesics, sedatives, and/or neuromuscular blocking agents which, remarkably, included 83 different agents. Analgesic agents were administered to 58.4% of children (38,776); of these, non-opioid analgesics were administered to 67.4% of children (26,149). The median duration for the administration of opioid analgesics was found to be 32 h (interquartile range; 7–92 h). Sedatives were administered to 39.8% of children (26,441) with a median duration of 23 h (with an interquartile range of 3–84 h), the most commonly administered example of which were benzodiazepines (73.4%; 19,426 children). Similar variation in clinical practice was demonstrated by Jenkins and colleagues in 2007, who published the results of an observational, prospective study involving 338 critically ill pediatric patients being cared for in 20 PICUs in the UK. The authors found total of 24 different analgesic and sedative agents were being used during the study with the agents used most in the sedative and analgesic classes being midazolam and morphine [7].

When Playfor and colleagues reviewed UK practice in 2003, the sedative agent administered most often was midazolam, alongside morphine and written clinical guidelines for the management of sedation in critically ill pediatric patients in this context were available in 45% of the units surveyed [8].

In a survey of 215 PICUs in 27 European countries, carried out by Daverio and colleagues in 2022, 71% of PICUs stated that they used clinical guidelines for sedation and analgesia. These guidelines were more frequently used in lower-volume PICUs with 450 admissions per year or less (77% vs. 63%, *p* = 0.028). The most popular drug combination was found to be fentanyl (51%) and midazolam (71%). Alpha-2 agonists were used infrequently as a primary agent, in only 18% of the PICUs, with dexmedetomidine being used more frequently than clonidine. Ketamine was used more often in PICUs with higher volumes of patients (16% vs. 2%, *p* = 0.000). A total of 81% of units carried out daily assessments of pain and 87% of units carried out daily assessments of sedation was reported by the majority of units. The most commonly used tool for pain assessment was the FLACC scale (used by 54% of units) and the most commonly used tool for sedation assessment was the COMFORT Behavioral score (48%) [9].

It has been demonstrated that sedation and analgesia clinical practice varies considerably by geography [10] and by specialty; infants who may be cared for in either Neonatal Intensive Care Units or PICUs have been shown to be likely to receive very different management [11].

Most critically ill children who need mechanically ventilated require a combination of multiple sedative and analgesic agents to maintain their comfort; Tillman and colleagues demonstrated that a mean of 2.58 ± 1.18 agents are required for each critically ill child during their critical care admission [12]. In this retrospective, single-center study of 130 critically ill children, just 17% of children were managed with one therapeutic agent, 36% of children were administered two therapeutic agents, 29% of children were administered three therapeutic agents, 12% of children were administered four therapeutic agents, and 6% of children were administered five or six therapeutic agents during their PICU stay. The dosing ranges of medications received by children in this study were extremely variable, with patient weight, age, and hospital length of stay all being significantly associated with sedation requirements. The older and heavier patients required more medication (on a per kg basis) to achieve the desired level of comfort. Mean overall infusion rates for fentanyl were 1.67 ± 0.81 μg/kg/h, for morphine 0.12 ± 0.08 mg/kg/h, and for hydromorphone 17.84 ± 13.4 μg/kg/h. For dexmedetomidine, the mean infusion rate was found to be 0.59 ± 0.28 μg/kg/h, while for midazolam, this value was found to be 0.14 ± 0.1 mg/kg/h.

As such ubiquitous agents, sedative and analgesic drugs are common contributors to adverse drug events in the pediatric critical care environment; Silva and colleagues have identified that sedative and analgesic agents are associated with 15.5% of adverse drug events in PICUs, with each event being associated with an increased length of stay [13].

It must be remembered that non-pharmacological interventions can play a significant role in maintaining the comfort of critically ill children. Such environmental factors can include family presence, reducing ambient noise, provision of music, sleep hygiene, early mobilization, and the use of virtual and augmented reality technology. Maximizing the presence of parents, guardians, and regular caregivers in the PICU during the provision of routine care and during invasive procedures can provide comfort to the child while also decreasing levels of parental distress and improving the quality of the patient experience. Several studies have demonstrated that personalized music intervention is feasible and helpful; in 2020, Liu and colleagues published a feasibility study of critically ill, mechanically ventilated children who were exposed to a music intervention, personalized to their taste [14]. Children in the intervention arm of the study listened to their favorite music for 60 min, three times a day. Children exposed to music had lower scores on the COMFORT Behavioral scale (15.7 vs. 17.6; *p* = 0.011) and better physiological outcomes, with lower heart rates (140 bpm vs. 144 bpm; *p* = 0.039), lower respiratory rates (40 bpm vs. 43 bpm; *p* = 0.036), lower systolic blood pressures (93 vs. 95 mmHg; *p* = 0.031), improved levels of oxygen saturation (96% vs. 95%; *p* < 0.001); it was found that the diastolic blood pressure was not significantly impacted (52 vs. 53 mm Hg; *p* = 0.11). Those patients exposed to music also benefitted from a shorter period of mechanical ventilation (148.7 vs. 187.6; *p* = 0.044) and a reduced duration of admission, but not significantly so (11.2 vs. 13.8; *p* = 0.071). The patients in the control arm required greater total doses of midazolam. Similarly, exposure to live music has been shown to be beneficial within the critical care environment [15,16]. Additionally, extended reality (XR) technology is being increasingly used in pediatric critical care medicine with suggested benefits including relief of pain, reduction in anxiety, and improvement in sleep and physiological parameters such as integer heart rate variability [17,18].

## 2. Clinical Guidelines

In response to concerns regarding the considerable variation in clinical practice, the first consensus clinical guidelines for providing analgesia and sedation to critically ill pediatric patients were developed and published by Playfor and colleagues in 2006 using a modified Delphi technique [19]. Twenty key recommendations were produced, with ten of these detailing analgesia practice and ten detailing sedation practice in critically ill pediatric patients. Particular emphasis was placed on non-pharmacological interventions including environmental factors, relaxation, distraction, promotion of sleep, and day/night orientation.

In more recent years, two significant consensus-based clinical guidelines for the provision of sedation and analgesia in critically ill children have been produced; in 2022, the Italian Society of Neonatal and Pediatric Anesthesia and Intensive Care (SARNePI) published their ‘Recommendations for analgesia and sedation in critically ill children admitted to intensive care unit’ [20], and also in 2022, Smith and colleagues published the ‘2022 Society of Critical Care Medicine Clinical Practice Guidelines on Prevention and Management of Pain, Agitation, Neuromuscular Blockade, and Delirium in Critically Ill Pediatric Patients With Consideration of the ICU Environment and Early Mobility’ [21]. The Italian group recommended a first-line strategy optimizing analgesia with opiates and suggesting alpha-2 agonists as the first-line sedatives, with benzodiazepines being viewed as secondary agents, with the administration of ketamine in cases of difficult analgesia/sedation. The Society of Critical Care Medicine (SCCM) guidelines included 44 different recommendations (14 of these were strong and 30 were conditional) along with five statements of good practice. They recommend that intravenous opioids are used as first-line analgesic agents for treating pain of moderate to severe intensity for critically ill children in this context, and that alpha-2 agonist agents are administered as the first-line sedative agents in critically ill children requiring mechanical ventilation.

## 3. Measuring Sedation

While there are many clinical scoring scales that have been reported in the medical literature, only relatively few of these have been thoroughly evaluated as to their applicability to critically ill children in the PICU. The COMFORT scale [22] has been used very frequently in research into sedation in the PICU and was recommended by the European Society of Pediatric and Neonatal Intensive Care (ESPNIC) for measuring sedation, in this context, since 2016 [23]. The COMFORT scale was first constructed in 1992 and comprises scales in eight domains involving the documentation of spontaneous movements, calmness, any facial tension, alertness, respiratory activity, muscle tone, heart rate, and blood pressure. Follow-up studies demonstrated that the physiological variables (heart rate and blood pressure) were not required and subsequently, an abbreviated version, which became known as the ‘behavioral’ COMFORT-B score, which utilized the other six domains, became very popular and has been extensively validated and adopted into routine practice.

There is increasing interest in the applicability of processed electroencephalography (EEG) monitoring for assessing the depth and quality of sedation within critical care with many devices now being available. However, the utility and optimal deployment of this type of monitoring remains unclear [24].

## 4. Daily Interruption of Sedation

In 2000, Kress and colleagues published the results of a randomized, controlled trial which included 128 mechanically ventilated, critically ill adults who were being sedated with continuous infusions of sedative agents [25]. Patients who underwent a daily interruption of sedative agents required mechanical ventilation for a median duration of 4.9 days, which was significantly less than the 7.3 days observed in those receiving conventional treatment (*p* = 0.004). The group with a daily interruption of sedative agents also benefitted from a significantly reduced duration of admission in critical care with 6.4 days of admission, much shorter than the 9.9 days of admission observed in those receiving conventional treatment (*p* = 0.02). However, concerns about daily interruption of sedation including patient discomfort, treatment interference and increased clinician workload, led to further work in this area; the results of a randomized, controlled trial facilitated by the SLEAP Investigators subsequently demonstrated that a daily interruption of sedative agents in 65 adult patients in 14 Canadian and 2 US intensive care units led to no difference in median time to successful extubation, no difference in critical care or hospital stay, but increased nursing workload [26]. A similar study of daily interruptions of sedative infusions compared to conventional continuous infusions in 102 mechanically ventilated pediatric patients, published by Gupta and colleagues in 2012, suggested the length of mechanical ventilation in the group with daily interruptions of sedative infusions was markedly shorter than that seen in the conventionally treated group (7.0 ± 4.8 days vs. 10.3 ± 8.4 days; *p* = 0.021). It was also noted that the median duration of PICU stay was significantly less in the interrupted sedation group as compared to the continuous sedation group (10.7 days vs. 14.0 days; *p* = 0.048) [27]. In 2015, Curley and colleagues published the results of the RESTORE study, a cluster-randomized trial of 2449 children carried out in 31 PICUs in the US to investigate the impact of a goal-directed sedation protocol, implemented by nursing staff, compared to conventional treatment [28]. There was no statistically significant difference in the length of mechanical ventilation when the two groups were compared; the median ventilation duration (with interquartile ranges) were 6.5 days (4.1–11.2) for the intervention and 6.5 days (3.7–12.1) for the conventional treatment group. The investigators identified that there was no statistically significant difference in sedation-related adverse incidents when comparing the two groups. Sedation-related adverse incidents were predefined as inadequate pain control and sedation provision, occurrence of iatrogenic withdrawal syndrome, and significant treatment interference. Blackwood and colleagues conducted a pragmatic cluster randomized clinical trial with a stepped-wedge implementation design, that was conducted in 18 PICUs in the UK [29]. A total of 4688 children were managed according to a sedation and ventilator liberation protocol intervention that included an assessment of the level of sedation, a daily evaluation for the readiness to carry out a trial of spontaneous breathing, a trial of spontaneous breathing to assess the potential for ventilator liberation, and ensuring that every 24 h there was a review of sedation and an assessment of readiness for ventilator liberation and to set individual targets for the patients. It was demonstrated that implementation of the protocol was associated with a significantly shorter period of time to successful extubation when contrasted with conventional treatment. This was found to be 64.8 h and 66.2 h, respectively, with an adjusted median difference of −6.1 h (the interquartile range was from −8.2 to −5.3 h); the adjusted hazard ratio was 1.11 (95%CI, 1.02 to 1.20), *p* = 0.02: Whilst statistically significant, a 6 h reduction in the duration of mechanical ventilation is of uncertain clinical significance.

It is for this reason that the latest SCCM guidelines state that daily interruption of continuous infusions of sedative agents as part of nurse-led protocolized sedation is not recommended due to lack of significant benefits or demonstrably better outcomes.

Intensive Care Unit Liberation is a philosophy that promotes wakefulness among critically ill patients, allowing for them to be interactive and as physically mobile as possible to reduce the burden of critical care-acquired morbidity. Survivors of pediatric critical illness have been demonstrated to suffer from significant morbidity in their physical health, their cognitive function, and their psychosocial performance; these factors have been associated with delayed recovery times, the increased consumption of various healthcare resources, overall impairment of function, and reduction in the quality of life when measured by different metrics.

The bundle of care known as ABCDEF includes the **A**ssessment, prevention, and management of pain; the use of **B**oth spontaneous awakening and spontaneous breathing trials; optimal **C**hoices of analgesic and sedative agents; **D**elirium assessment, prevention, and management; optimizing **E**arly mobility and exercise; and maximizing **F**amily engagement and patient/family empowerment. It is at the heart of the ICU Liberation collaborative, facilitated by the Society of Critical Care Medicine. This evidence-based, multifaceted, six-step program was constructed with the aim of actively liberating adult patients from restrictive interventions that had been instigated in the critical care unit. Carrying forward this approach into the PICU environment is challenging given the lack of definitions around early mobilization, age-related variations, developmental status, comorbidities, and diagnostic variation. The PICU Up! initiative undertaken at Johns Hopkins Hospital in the US is notable as the first structured, multi-disciplinary program of early mobilization demonstrated to improve activity levels in children within a Pediatric Critical Care Unit [30]. Initially, the quality improvement program focused on the introduction of bundles of care to improve the hygiene of sleep and to prevent delirium, while the subsequent PICU Up! trial was a multicenter cluster-randomized trial with a stepped-wedge design used to assess the effectiveness of the study protocol. In the baseline phase, the trial recruited 1196 patients, and following the introduction of the intervention, 1076 patients were recruited; this led to a significant increase in compliance with the bundle. In a review of 161 participating PICUs, Ista and colleagues demonstrated that only 15 (9%) managed to incorporate all of the six ABCDEF bundle components into daily practice [31]. The widespread adoption of PICU liberation programs has been hindered by mixed evidence of efficacy in PICU, the time- and resource-intensive nature of facilitating effective mobilization, the acknowledged heterogeneity of the PICU population compared to that seen in adult critical care, and a lack of confidence amongst clinical staff in mobilizing critically ill children [32]. In a 2019 survey of UK PICUs, Thompson and colleagues identified that limited resources and the lack of local and national clinical guidelines were significant barriers to adoption [33].

In an international study of 380 PICUs in 47 countries, Loberger and colleagues demonstrated considerable variation in international pediatric ventilation liberation practice and poor protocol implementation [34]. Similar difficulties have been encountered in promoting organizational compliance with delirium screening tools [35], and considerable educational initiatives are required to embed these developments into routine clinical practice [36,37]. It is widely accepted, however, that optimizing the choice and dosing of sedatives and analgesic agents together with the appropriate rapid weaning of mechanical ventilatory support are important elements in optimizing outcomes among the survivors of critical illness in childhood [38]. PICU-STARS was designed as a single-center ‘before-and-after’ trial and implementation study that was constructed to assess if this sort of multi-factorial, nurse-implemented PICU liberation structure of care delivery can be carried out in a pediatric critical care environment and be effective in reducing iatrogenic problems related to PICU within a mixed quaternary center [39].

## 5. Withdrawal

The likelihood of withdrawal (which is quoted as around 35% for all ventilated, sedated PICU patients) is directly related to the overall quantity of the sedative agents administered to the patient. Symptoms of withdrawal include central nervous system irritability (poor sleep pattern, tremor, irritability, hallucinations, or convulsions), gastrointestinal disturbance (which can include vomiting, non-infective diarrhea, and abdominal pain) and autonomic disturbances (such as sweating, pyrexia, yawning, hiccups, shivers, an increase in airway secretions, increasing heart rate, increasing respiratory rate, and high blood pressure). Withdrawal has long been associated with the use of opioids and over more recent years it has become clear that almost all therapeutic agents conferring sedation and analgesia are also associated with the induction of tolerance and iatrogenic withdrawal; these agents include midazolam, clonidine, dexmedetomidine, and propofol.

It appears that opioid tolerance occurs earlier in younger pediatric patients and that the development of tolerance may be worsened by underlying neurological insults and also be associated with short-acting opioids, which have a high affinity for opioid receptors.

There are two validated scoring tools for the quantification of iatrogenic withdrawal available for PICU practice: the Withdrawal Assessment Tool-1 (WAT-1) [40] and the Sophia Observation Score (SOS) [41]. The WAT-1 consists of 11 domains and takes around 7 min for completion. The SOS tool was developed following a multi-parameter assessment of simultaneously occurring factors between pediatric patients who had been separated into two cohorts: one group who were thought to be suffering from iatrogenic withdrawal whilst receiving a reducing schedule of sedative agents, and one group who were not thought to be suffering from iatrogenic withdrawal whilst receiving a reducing schedule of sedative agents. The cohorts were determined by a team of experienced critical care doctors and nurses. It has been suggested that SOS scores are most useful in identifying those patients who will not go on to display frank features of iatrogenic withdrawal but, in keeping with other similar tools, may be less useful in identifying patients who will go on to display frank features of iatrogenic withdrawal. It has also been identified that other neurophysiological disturbances, including discomfort, pain, and delirium, can complicate the assessment of iatrogenic withdrawal.

## 6. Delirium

Delirium is recognized as being an acute neurological dysfunction in the context of critical illness and is characterized by a fluctuating pattern of disruption in awareness and cognition. Delirium can be identified in around 40% of children who have been admitted to the pediatric critical care unit for 6 days or more. Recognized risk factors for the development of delirium include mechanical ventilation, benzodiazepine administration, narcotic administration, use of physical restraint, and exposure to vasopressors and anti-epileptic agents. Children with developmental delay have been shown to have a 3.5 times greater likelihood of having a diagnosis of delirium in PICU. Delirium can lead to longer periods of mechanical ventilation and prolonged PICU admission and is a strong and independent predictor of mortality. It is associated with a significant financial burden on healthcare systems and has been associated with an 85% increase in PICU costs.

Administration of sedative and neuromuscular blocking agents, and the occurrence of withdrawal symptoms and delirium are also significant risk factors for the development of the so-called post-intensive care syndrome and also for significant neuromuscular weakness due to polyneuropathy or myopathy known as ‘ICU-acquired weakness’. Pediatric delirium has been linked to a subsequent clinically important deterioration in various health-related quality of life scores.

Validated assessment tools are available, specifically for critically ill children. The pCAM-ICU is a tool constructed for use with young people over the age of 5; it is a cognitively focused, interactive tool. Another delirium tool, the Cornell Assessment of Pediatric Delirium (CAPD), is an observational device that has been constructed for use with children of any age and has also been validated for deployment with those displaying non-normative developmental skills [42]. The Preschool Confusion Assessment Method (psCAM-ICU) has been constructed more recently to allow for the assessment pediatric delirium; it has been validated to be used in young people between the ages of 6 months and 5 years [43]. The construction and dissemination of the CAPD and psCAM-ICU devices are important developments that will provide clinical teams working in PICU the ability to screen for, monitor, and manage delirium in pediatric patients within critical care environments, even including children who are intubated and being mechanically ventilated.

## 7. Commonly Used Sedative Agents

Typical doses of commonly used sedative agents for critically ill children are shown in Table 1.

### 7.1. Opioids

Morphine has played a pivotal role in medicine since its discovery by Friedrich Wilhelm Adam Sertürner in 1805 and has been a cornerstone of analgesia for children since the development of organized PICUs in the 1950s and 1960s.

It has long been recognized that prolonged administration of opioids induces tolerance with a requirement to administer increased doses of the agent to generate the same clinical impact [44]. This effect may be due to either what is known as ‘tolerance’, characterized by a desensitization of the μ-opioid receptor complexes that occurs following repeated stimulation, or what is known as ‘tachyphylaxis’, characterized by compensatory physiological changes, such as up-regulation of antagonist pathway systems like the *N*-methyl-D-aspartate (NMDA) pathway. Both of these mechanisms mean that if the opioid is suddenly removed, withdrawal symptoms can develop.

Morphine is the only poorly lipid-soluble opioid in common use; it produces active metabolites that are dependent on adequate renal elimination; it is converted in the liver to morphine-6-glucuronide, a product that has significant activity at opioid receptors, and morphine-3-glucuronide. In preterm infants, the renal clearance of morphine fails to normalize at term. Maturation develops over a longer trajectory; therefore, morphine should be administered to preterm infants in modest doses to take account of this pharmacokinetic variation.

Morphine administration may result in the liberation of large quantities of histamine and also inhibit sympathetic efferent responses that would normally compensate for this; therefore, the vasodilatation generated by morphine can produce clinically important falls in blood pressure, especially following the administration of bolus doses.

Fentanyl, first synthesized in 1960 by Paul Janssen, is a man-made opioid agent that has 100 times the pain-relieving effects of morphine. Fentanyl is a very lipid-soluble agent, which explains its rapid onset following administration. The administration of fentanyl generally liberates smaller amounts of histamine than is seen with morphine, and because of this, fentanyl is associated with more cardiovascular stability. However, fentanyl can lead to a reduction in cardiac output by decreasing the heart rate. When given intravenously, fentanyl rapidly redistributes into peripheral compartments, which means the drug has a relatively short half-time. With extended infusions, fentanyl accumulates within peripheral compartments, leading to a significant prolongation of the context-sensitive half-time, meaning that tolerance can become apparent quickly. Metabolism of fentanyl occurs almost completely within the liver and clearance is therefore dependent upon the blood flow to the liver. As there are no active metabolites of fentanyl; allergy to morphine confers no cross-sensitivity to fentanyl.

Remifentanil is a newer man-made opioid, derived from phenylpiperidine; its mechanism of action is solely as an agonist to μ-receptors. It is equally potent as fentanyl, with similar cardiorespiratory effects to other opioids but with unique properties. Remifentanil is notable for its extremely short half-time of only 3 min in patients of all ages, as it is metabolized by esterases in the plasma and the tissues; as such, it has an exceptionally small volume of distribution. The physiological effects of remifentanil reliably wear off very rapidly, even when given by a prolonged infusion, meaning that it has an extremely short context-sensitive half-time. Remifentanil infusions are being used increasingly frequently to facilitate pain relief in critically ill children; however, tolerance to this agent can develop very quickly with prolonged administration by continuous infusion and is a relatively high-cost therapeutic agent.

Benefits: Morphine improves ventilator synchrony, blunts catecholamine surges, and when used in an analgesia-first paradigm can reduce the requirement for other hypnotic agents. Fentanyl confers rapid procedural analgesia, minimal histamine release, and cardiovascular stability. Remifentanil can enable fast, predictable wake-up times to facilitate neurological examinations or when extubation needs to occur at specific times.

Risks: All opioids carry the risk of respiratory depression, constipation, and tolerance. The risks of oversedation can be mitigated with the use of regular sedation scoring systems with nurse-driven titration of analgesic agents. Opioid stewardship programs employ multimodal adjuvants (such as paracetamol, non-steroidal anti-inflammatory agents, and low-dose ketamine) to significantly reduce cumulative doses of administered opioids. Structured tapering of opioid doses by 10–20% increments of the original dose daily or rotation to enteral opioids may help prevent iatrogenic withdrawal syndrome when infusions are administered for longer than five days.

### 7.2. Benzodiazepines

Benzodiazepines are active at gamma-aminobutyric acid (GABA) receptors, which make up the dominant inhibitory neurophysiological system within the human central nervous system. In the sedation of critically ill children, the most frequently used benzodiazepines are midazolam, lorazepam, and diazepam.

Midazolam was initially synthesized in the mid-1970s and was rapidly adopted into ICUs as a potent, short-acting, relatively water-soluble benzodiazepine that at plasma pH converts into an un-ionized form that crosses the blood–brain barrier rapidly and has the shortest elimination half-time of the benzodiazepines. It produces antegrade amnesia without impairing the retrieval of previously learned information. Following a single bolus injection, midazolam takes 5–10 min to reach maximum sedative effect and the sedation impact is maintained for between 30 and 120 min. When midazolam is administered by continuous infusion, the length of action is notably prolonged, and with extended administration, the latent sedative effects may continue for 48 h after discontinuation of the drug.

Cytochrome P450 isoenzyme 3A4 hydroxylation metabolizes midazolam to 1-hydroxymidazolam and to 1,4-dihydroxymidazolam, which then undergoes further glucuronidation. CYP3A4 is the most common cytochrome P450 enzyme within the liver and the gut, and it metabolizes more than 50% of all medications. Glucuronidation of 1-hydroxymidazolam by UDP-glucuronosyltransferases generates inactive metabolites that are excreted in the urine. When active metabolites accumulate, for instance, in patients suffering from renal failure, very extended sedative duration of action can be seen. Similarly prolonged sedation can be seen when there is competition for substrate for CYP3A4 after the co-administration of certain drugs, such as erythromycin. Prolonged sedation can also be seen with inflammation-induced suppression of CYP3A4, which is driven by inflammatory cytokines such as interleukin-6.

Midazolam has been the most commonly administered sedative agent for prolonged intravenous administration in PICUs over the last 30–40 years. The most common drawbacks associated with midazolam are the emergence of tolerance, dependence, and iatrogenic withdrawal phenomena following subsequent weaning or discontinuation of the agent. Hypotension may be seen, especially after the administration of a bolus dose of midazolam where there is co-existing hypovolemia. Midazolam has been demonstrated to be less effective when it is administered by continuous infusion to infants compared to older children.

Benefits: Provides anxiolysis, antegrade amnesia, and muscle relaxation. Intranasal midazolam can facilitate procedures and diagnostic imaging without intravenous access.

Risks: Observational studies have linked benzodiazepines to prolonged mechanical ventilation and the development of delirium. Consequently, modern guidelines advocate benzodiazepine-sparing bundles and the preferential use of α-2 agonists as first-line agents for ongoing sedation [20,21].

### 7.3. Ketamine

Ketamine has been used in clinical practice in critical care since the 1960s; it is a fast-acting general anesthetic with both sedative and analgesic properties that offers value as an adjunct for sedation in mechanically ventilated PICU patients. Ketamine blocks glutamate by antagonizing *N*-methyl-D-aspartate (NMDA) receptors but also affects a variety of other cellular signaling pathways. It induces a dissociative state, provides effective anesthesia, and also has antidepressant effects. Ketamine maintains pulmonary compliance while reducing airway resistance, meaning it has demonstrated benefits in children with severe bronchospasm. Unlike other sedative agents, ketamine has mostly beneficial effects on the cardiovascular system; it tends to increase catecholamine concentrations through a combination of release and reuptake blockade, which produces a modest elevation in heart rate and blood pressure and boosts cardiac output. There are concerns that in catecholamine-depleted states, ketamine administration may lead to hypotension when given as a bolus dose. Ketamine has been associated with emergence phenomena, with vivid dreams and hallucinations being reported. Sub-anesthetic infusions of ketamine have been shown to reduce opioid requirements by 30–50% and mitigate opioid-induced hyperalgesia [45].

Benefits: Analgesia and sedation while preserving respiratory drive; hemodynamic stability; potent bronchodilation; during controlled ventilation, ketamine can reduce intracranial pressure while raising central perfusion pressure. NMDA blockade can terminate seizures resistant to GABAergic drugs without respiratory compromise. Continuous ketamine has been integrated into burns unit sedation protocols, allowing for reductions in benzodiazepine and opioid exposure by up to 40% [46].

Risks: Sympathomimetic surges; sialorrhea (drooling or hypersalivation), which can be mitigated with glycopyrrolate or atropine. Laryngospasm risk is negligible at sub-dissociative doses. Emergence agitation, vivid dreams, and hallucinations in school-age children and adolescents. Mild, reversible transaminase elevation with prolonged use for over 5 days. Can potentiate hypotension when administered in combination with propofol or high-dose dexmedetomidine. Apnea can occur in children, particularly in neonates, after rapid intravenous bolus dosing of ketamine but is a rare phenomenon, occurring in 0.3% of cases [47,48]; slower intravenous bolus administration over 60 s eliminates this problem [49].

### 7.4. Alpha-2 Agonists

Clonidine was introduced into clinical practice in 1966 as a centrally acting anti-hypertensive agent. It has since become popular as a low-cost intravenous sedative agent that preserves respiratory drive. The α-2 agonists have effects at multiple areas where α-2 receptors are found. These include sympathetic nerve endings, presynaptically, mediating sympatholysis; in areas like the substantia gelatinosa, modulating the release of substance P relieving pain; and also at the locus coeruleus, facilitating both analgesia and sedative effects. These agents also potentially act on the nucleus ambiguus as well as the vagal nerve, via the dorsal motor nucleus, leading to stimulation of the parasympathetic system.

In children, the clearance of clonidine is dependent upon kidney function, with around half being excreted unchanged by the kidney and half being transformed in the liver. For smaller infants, clonidine clearance depends on renal maturity. When administered by extended infusions, the context-sensitive half-time of clonidine can increase by 100% because of its high lipid solubility and due to accumulation within peripheral physiological compartments.

The use of clonidine accelerated after the 2014 SLEEPS randomized clinical trial [50], which showed that clonidine infusions were as effective as midazolam in critically ill children yet was cheaper and associated with fewer iatrogenic withdrawal symptoms. This led many units to adopt intravenous and enteral clonidine as first-line sedative and weaning agents.

Dexmedetomidine is an α-2 agonist that, when compared to clonidine, has an eight-fold increased affinity for α-2 receptors. While clonidine has a half-time of 12–24 h, dexmedetomidine has a half-time of only 2–3 h. Dexmedetomidine is metabolized to 3-hydroxy-dexmedetomidine, which is believed to carry just 0.5% of the activity of the parent compound at α-2 receptors, this contributes to the safety profile of dexmedetomidine. It has demonstrated sedative, analgesic, and anxiolytic effects and is well tolerated in critically ill children [51]; the Baby SPICE study confirmed that a dexmedetomidine-first, benzodiazepine-sparing sedation pathway was feasible, safe, and effective [52].

Modern sedation guidelines advocate benzodiazepine-sparing bundles and the preferential use of α-2 agonists as first-line agents for ongoing sedation [20,21].

Benefits: Minimal to little effect on respiratory drive, facilitates early spontaneous breathing tests, opioid and benzodiazepine reduction, patient can remain rousable, fewer days of agitation, and reduced iatrogenic withdrawal syndrome.

Risks: Bradycardia (2–10%) and hypotension require low starting rates and atropine readiness. Rebound hypertension can be avoided by infusion tapering or oral clonidine bridging.

### 7.5. Volatile Anesthetics

Volatile anesthetic agents have been used sporadically to provide sedation to critically ill patients in critical care units since the 1980s. Early experience demonstrated the consistent finding of sedation quality that was at least as good as standard intravenous agents, but with much more rapid wake-up and extubation times [53].

Early use of volatile agents was hampered by the practical complexity of delivery and scavenging; this meant that less capable anesthetic ventilators needed to be used, or ad hoc scavenging systems had to be put together. Case series published in the 1990s demonstrated that prolonged administration of volatile agents to critically ill children was practical and feasible and delivered the same benefits of high quality sedation and rapid wake-up times following discontinuation [54]. A significant breakthrough was the development of inline vaporizer the anesthetic conservation device, AnaConDa™ (which is currently being rebranded as the Sedaconda ACD). Proposed in the mid-1990s by Louis Gibeck, who was the developer of the original disposable anesthetic humidified moisture exchanger device, the AnaConDa™ is a single-use, disposable, inline vaporizing device designed to deliver volatile anesthetics (isoflurane or sevoflurane) to patients on mechanical ventilation, using existing critical care ventilators and standard syringe pumps. During the 2000s, use of the AnaConDa™ was described in anesthesia, adult critical care, and PICUs. The AnaConDa-S™, with only a 50 mL dead space, was released in 2017, this allowed for the device to be used in its standard configuration on smaller children.

In 2021, Meiser and colleagues demonstrated that isoflurane was not inferior to sedation with propofol given by infusion to critically ill adult patients, finding that use of inhaled isoflurane was associated with reduced opioid requirements, facilitated spontaneous breathing, and led to faster, more predictable emergence from sedation [55]. The results of a similar international, multicenter trial, the IsoCOMFORT study, conducted in critically ill children, are currently in press.

Benefits: Dependable route of drug administration and elimination. Little metabolism. Rapid onset and offset; shorter wake-up times. Less variability in dose–response effect. Easily titratable depth of sedation. Bronchodilation, volatile agents have been shown to be life-saving in cases of acute severe asthma. Potential organ protective properties—isoflurane and sevoflurane have been demonstrated to have cytoprotective effects in several organ systems through various mechanisms, including the activation of protective signaling pathways by modulating inflammation.

Risks: Rare cases of malignant hyperthermia (1:62,000). Hypotension. Rapid wakening during device disconnection, such as for physiotherapy. Reversable neurological phenomenon including pupillary changes and clonus.

Greenhouse and environmental effects: Volatile agents are known to have a significant greenhouse effect with global warming potential [56]. During anesthesia and critical care use, volatile agents are usually scavenged to avoid potential occupational exposure; however, they are eventually released to the outside atmosphere. It is believed that the estimated total greenhouse contribution by these anesthetic agents is 0.01–0.1% [57,58]. It should be noted that desflurane has the most significant global warming potential of the commonly used volatile sedative agents (although this agent cannot be given via the AnaConDa™ device), five times that of isoflurane.

### 7.6. Propofol

Propofol, 2,6 Di-isopropylphenol, was first synthesized in 1973 by Imperial Chemical Industries; this was achieved by replacing hydrogen in the solvent 1,3 Di-isopropylbenzene with hydroxy groups. Propofol, prepared as a lipid emulsion was approved for clinical use in 1986 both in New Zealand and in the United Kingdom. In 1989, propofol, under the trade name Diprivan, was approved by the FDA. The favorable pharmacokinetic profile of propofol, with remarkably short onset and offset times and relative cardiovascular stability, have made it the world’s most frequently used intravenous hypnotic agent, and it is currently used in hundreds of millions of surgeries every year.

The first case of death attributed to the now so-called propofol infusion syndrome (PRIS) occurred in Denmark in 1990, and a warning was issued by the Danish Side-effects Committee regarding the use of propofol when given by infusion to children. In 1992, a report by Parke and colleagues generated significant, widespread concern about the safety of continuous propofol infusions in children. The authors reported on the deaths of five children aged between 4 weeks and 6 years who were mechanically ventilated in the PICU due to severe infections of the respiratory tract. The patients in this study were all sedated with high-dose propofol infusions, and developed metabolic acidosis, hyperlipidemia, hepatomegaly, bradyarrhythmias, and, ultimately, progressive cardiac failure [59]. It is believed that the development of PRIS is related to the inhibition of intracellular energy production within mitochondria by propofol.

Propofol is not recommended for long-term sedation of critically ill children in any recognized clinical guideline. When Playfor and colleagues published the first UK national consensus guidelines on providing analgesia and sedation in the PICU in 2006, an explicit recommendation was made, stating that ‘Propofol should not be used to provide continuous sedation in critically ill children’ [19].

In the 2022 US SCCM Clinical Practice Guidelines it is suggested that propofol sedation, given at low doses and for less than 48 h continuously, may be safe and could be a useful adjunct during the time around extubation to allow for the reduction in other analgesic and sedative agents to facilitate extubation [21].

## 8. Future Innovation

Volatile anesthetic agents are likely to be used increasingly frequently in this context as evidence of their benefits continue to be illustrated. Xenon is a noble gas that has been used sporadically as a sedative agent in critical care settings due to its unique properties with rapid onset and offset of action, minimal cardiovascular effects, and potential neuroprotective benefits. Currently, xenon is prohibitively expensive but advances in closed-circuit ventilation systems with agent recycling will make its use more feasible. Ciprofol is a γ-aminobutyric acid receptor agonist that has been recently developed. Ciprofol is a short-acting sedating agent that has greater potency than propofol; in 2022, the drug was approved for the provision of sedation in critically ill patients undergoing mechanical ventilation [60]. Remimazolam is a novel ultra-short-acting benzodiazepine, showing promise for sedation in critical care due to its rapid onset, organ-independent metabolism, and quick recovery time [61]. Sufentanil is a synthetic opioid that has between 5 and 10 times the potency of fentanyl with rapid onset and a prolonged sedative effect. FDA-approved in 1984, sufentanil has been used to provide long-term sedation in the intensive care unit. The pharmacodynamic profile of sufentanil with prompt redistribution and metabolism should mean that sedation with this agent is rapidly reversible even after an extended continuous infusion [62]. Alfaxalone (3α-hydroxy-5α-pregnane-11,20-dione) is a man-made neuroactive steroid that has been used in veterinary practice since the 1970s as a rapid-onset intravenous anesthetic agent which has a wide safety margin between effective and toxic doses [63]. Alfaxalone is believed to be neuroprotective and free from neurotoxic activity. Brexanolone is a GABA-A modulator, a proprietary, aqueous formulation of the neuroactive steroid allopregnanolone [64]. It has been used in post-partum depression, as a treatment for super-refractory status epilepticus, and shows promise as a sedative for critically ill patients. Currently, there is extremely limited experience of using these novel and relatively novel agents in critically ill children.

## Figures and Tables

**Table 1:** Recommended drug dosages.

Drug	Recommended Continuous Infusion Dose	Recommended Bolus Dose
Morphine	0–60 mcg/kg/h	50–100 mcg/kg
Fentanyl	0.5–6 mcg/kg/h	0.25–1 mcg/kg
Remifentanil	0.1–2 mcg/kg/min	0.25–1 mcg/kg
Midazolam	30–300 mcg/kg/h in children < 33 kg 0.5–10 mg/kg/h in children > 33 kg	100 mcg/kg
Ketamine	10–45 mcg/kg/min	0.5–2 mg/kg
Clonidine	0.5–2 mcg/kg/h	2–6 mcg/kg
Dexmedetomidine	0.2–0.7 mcg/kg/h	0.5–1 mcg/kg
Propofol	0.3–4 mg/kg/h	2.5–4 mg/kg
